# Ruxolitinib for the treatment of inadequately controlled polycythemia vera without splenomegaly: 80-week follow-up from the RESPONSE-2 trial

**DOI:** 10.1007/s00277-018-3365-y

**Published:** 2018-05-27

**Authors:** Martin Griesshammer, Guray Saydam, Francesca Palandri, Giulia Benevolo, Miklos Egyed, Jeannie Callum, Timothy Devos, Serdar Sivgin, Paola Guglielmelli, Caroline Bensasson, Mahmudul Khan, Julian Perez Ronco, Francesco Passamonti

**Affiliations:** 1 Department of Hematology, Oncology, Hemostaseology and Palliative Care, Johannes Wesling Clinic, Minden, Germany; 2 Department of Hematology, Ege University Medical Faculty, Izmir, Turkey; 3 Institute of Hematology “Seràgnoli”, St. Orsola-Malpighi Hospital, Bologna, Italy; 4 Department of Hematology, Città della Salute e della Scienza di Torino, Turin, Italy; 5 Hematology Department of Somogy County, Kaposi Mor Teaching Hospital, Kaposvar, Hungary; 6 Department of Transfusion Medicine and Tissue Banks, Sunnybrook Health Sciences Centre, Toronto, ON Canada; 7 Department of Hematology, University Hospitals Leuven and Laboratory of Experimental Transplantation, Department of Microbiology and Immunology, KU Leuven, Leuven, Belgium; 8 Department of Hematology, Dedeman Stem Cell Transplantation Hospital, Erciyes University, Kayseri, Turkey; 9 CRIMM, Center for Research and Innovation of Myeloproliferative Neoplasms, AOU Careggi, Department of Experimental and Clinical Medicine, University of Florence, Florence, Italy; 10 Novartis Pharma S.A.S, Rueil-Malmaison, France; 11 Novartis Pharmaceuticals Corporation, East Hanover, NJ USA; 12 Novartis AG, Basel, Switzerland; 13 Department of Hematology, University of Insubria, Varese, Italy

**Keywords:** Chronic myeloproliferative disorders, JAK inhibitor, Polycythemia vera, Ruxolitinib

## Abstract

RESPONSE-2 is a phase 3 study comparing the efficacy and safety of ruxolitinib with the best available therapy (BAT) in hydroxyurea-resistant/hydroxyurea-intolerant polycythemia vera (PV) patients without palpable splenomegaly. This analysis evaluated the durability of the efficacy and safety of ruxolitinib after patients completed the visit at week 80 or discontinued the study. Endpoints included proportion of patients achieving hematocrit control (< 45%), proportion of patients achieving complete hematologic remission (CHR) at week 28, and the durability of hematocrit control and CHR. At the time of analysis, 93% (69/74) of patients randomized to ruxolitinib were receiving ruxolitinib; while in the BAT arm, 77% (58/75) of patients crossed over to ruxolitinib after week 28. No patient remained on BAT by week 80. Among patients who achieved a hematocrit response at week 28, the probability of maintaining response up to week 80 was 78% in the ruxolitinib arm. At week 80, durable CHR was achieved in 18 patients (24%) in the ruxolitinib arm versus 2 patients (3%) in the BAT arm. The safety profile of ruxolitinib was consistent with previous reports. These data support that ruxolitinib treatment should be considered also as a standard of care for hydroxyurea-resistant/hydroxyurea-intolerant PV patients without palpable splenomegaly.

**Electronic supplementary material:**

The online version of this article (10.1007/s00277-018-3365-y) contains supplementary material, which is available to authorized users.

## Introduction

Polycythemia vera (PV) is a myeloproliferative neoplasm characterized by an abnormal increase in red blood cell mass due to an activating mutation in the *Janus kinase 2 (JAK2)* gene [1]. Approximately 40% of patients with PV present with an increase in white blood cell (WBC) and platelet counts [2]. Patients with PV have a substantial symptom burden [3], a high risk of vascular complications [4] and progression to myelofibrosis (MF) or acute myeloid leukemia (AML), and a shortened life expectancy [5, 6]. Splenomegaly is often seen in a subset of patients (approximately 30%) with PV; it is primarily responsible for the presence of abdominal symptoms [3] and is a predictor of shortened survival in these patients [3].

The therapeutic management of PV is aimed to alleviate the symptom burden, prevent the first occurrence and/or recurrence of thromboembolic events, and prevent the disease transformation to MF or AML [7]. The results from the Cytoreductive Therapy in Polycythemia Vera (CYTO-PV) study demonstrated that controlling the hematocrit (HCT) level below 45% was associated with a fourfold reduction in the rates of major thrombotic events and cardiovascular deaths [8]. In this study, hydroxyurea (HU) and phlebotomy were able to achieve a reduction in the rate of cardiovascular death and major thrombosis by controlling the HCT < 45%. However, no effect on the symptom burden was seen in patients, even after the long-term conventional aggressive therapy in CYTO-PV study [9]. It is well known that nearly a quarter of patients discontinue the first-line therapy (HU or interferon) due to the development of resistance or intolerance to treatment [10–12].

Ruxolitinib, a JAK1/2 inhibitor, has demonstrated a superior and durable response versus the best available therapy (BAT) in controlling HCT and improving splenomegaly and symptoms in patients with PV who were inadequately controlled with HU [13, 14]. Ruxolitinib was approved by the United States Food and Drug Administration (FDA) and the European Medicines Agency (EMA) for treating patients with PV who have an inadequate response to or are intolerant of HU based on the results from the primary analysis of the RESPONSE study [15]. A subgroup analysis from the RESPONSE study showed that the degree of splenomegaly at baseline is not a determinant of HCT control or spleen volume reduction by ruxolitinib [16]. This was further confirmed by the findings from the randomized, phase 3b RESPONSE-2 study in patients with PV who have an inadequate response to or unacceptable side effects from HU and have a nonpalpable spleen. In the RESPONSE-2 study, ruxolitinib was superior to BAT in providing control of HCT control at week 28 (primary end point; 62 versus 19% patients in the ruxolitinib versus BAT arm; *P* < 0.0001), inducing complete hematological remission (CHR), and improving disease-associated symptoms, regardless of absence of splenomegaly in the study patient population [17]. The present preplanned analysis of the RESPONSE-2 study was conducted to evaluate the durability of efficacy and safety of ruxolitinib after all patients completed the visit at week 80 or discontinued the study.

## Methods

### Study design

RESPONSE-2 is a prospective, randomized, open-label, multicenter, phase 3b study assessing the efficacy and safety of ruxolitinib versus BAT in patients with PV without splenomegaly who are resistant to or intolerant of HU. The study design and patient eligibility criteria have been described previously [17]. Patients were recruited from 48 hospitals or clinics across 12 countries. Patients were eligible if they had PV according to WHO criteria, were aged 18 years or older, had an Eastern Cooperative Oncology Group (ECOG) performance status of 0 to 2, had no palpable splenomegaly, had no previous treatment with JAK inhibitors, and were phlebotomy-dependent (a HCT between 40 and 45% achieved with phlebotomy within 14 days before randomization was required). Eligible patients with HCT > 45% before randomization entered a HCT control period to ensure that their HCT was similar and controlled at study initiation. Eligible patients also had to meet the definition of HU resistance (inadequate response to HU treatment) or intolerance (unacceptable side effects from HU treatment) according to modified European Leukemia Net (ELN) criteria [18].

### Procedures

Patients were randomly assigned (1:1) to receive either ruxolitinib or BAT by using a validated system that automated the random assignment of patient numbers to randomization numbers linked to the treatment groups; randomization was stratified by whether patients were resistant or intolerant to HU therapy. The starting dose of oral ruxolitinib was 10 mg twice daily and could be titrated up to a maximum of 25 mg twice daily (in 5 mg increments at each dose escalation). Single-agent BAT was chosen based on the physician’s discretion and included HU (at the maximum tolerated dose), interferon or pegylated interferon, pipobroman, anagrelide, approved immunomodulators, or observation without pharmacologic treatment. All randomized patients received a low dose of aspirin (75–150 mg/day), unless medically contraindicated.

From week 28 and until week 80, all patients randomized to BAT were allowed to cross over to the ruxolitinib arm if they did not meet the primary end point (i.e., HCT level > 45%, or if they received phlebotomy) or for safety-related reasons. If, by week 80, they did not cross over and still did not meet the criteria for crossover, these patients had to discontinue the study. The data cutoff for this 80-week analysis was September 26, 2016.

### Outcomes

The primary end point was the proportion of patients who achieved HCT control at week 28; HCT control was defined as the absence of phlebotomy eligibility between weeks 8 and 28, with phlebotomy eligibility occurring only once after randomization prior to week 8. Phlebotomy eligibility was defined as confirmed HCT level > 45% and at least 3 percentage points higher than baseline, or confirmed HCT level > 48%.

The key secondary end point was the proportion of patients achieving CHR (defined as HCT control, white blood cell count [WBC] < 10 × 10^9^/L, and platelet count ≤ 400 × 10^9^/L) at week 28. Other secondary end points were the durability of HCT control and CHR (i.e., the proportion of patients achieving HCT control and CHR at weeks 52 and 80); the change in phlebotomy eligibility over time, HCT level over time, spleen length as measured by palpation, ECOG status; transformation-free survival, overall survival, safety; and changes in patient-reported outcomes. Patient-reported outcomes were assessed from baseline to week 80 by several questionnaires: the Myeloproliferative Neoplasm Symptom Assessment Form Total Symptom Score (MPN-SAF TSS), the Pruritus Symptom Impact Scale (PSIS), the EuroQol-5D-5L (EQ-5D-5L), the Work Productivity and Activity Impairment (WPAI), and the Patient Global Impression of Change (PGIC). Adverse events (AEs) were assessed according to the National Cancer Institute Common Toxicity Criteria for Adverse Events version 4.03.

Please see the Online Supplementary Section for details related to statistical analyses included in this report.

## Results

### Patients and baseline characteristics

Patient baseline characteristics and primary results of the study have been reported previously [17]. In total, 149 patients were randomized to receive either ruxolitinib (*n* = 74) or BAT (*n* = 75). Overall, the baseline characteristics of patients were balanced between the treatment groups. The median ages in the ruxolitinib and BAT arms were 63.0 and 67.0 years, respectively. The median time periods since PV diagnosis were 6.5 and 6.7 years, and the median durations of previous HU therapy were 33.95 and 42.61 months in the ruxolitinib and BAT arms, respectively. At baseline, median HCT values were 43.0% in the ruxolitinib arm and 42.6% in the BAT arm. Approximately 78% of patients had received 2 or more phlebotomies within 24 weeks before screening in the ruxolitinib arm, compared to 76% in the BAT arm [17].

**Fig. 1:**
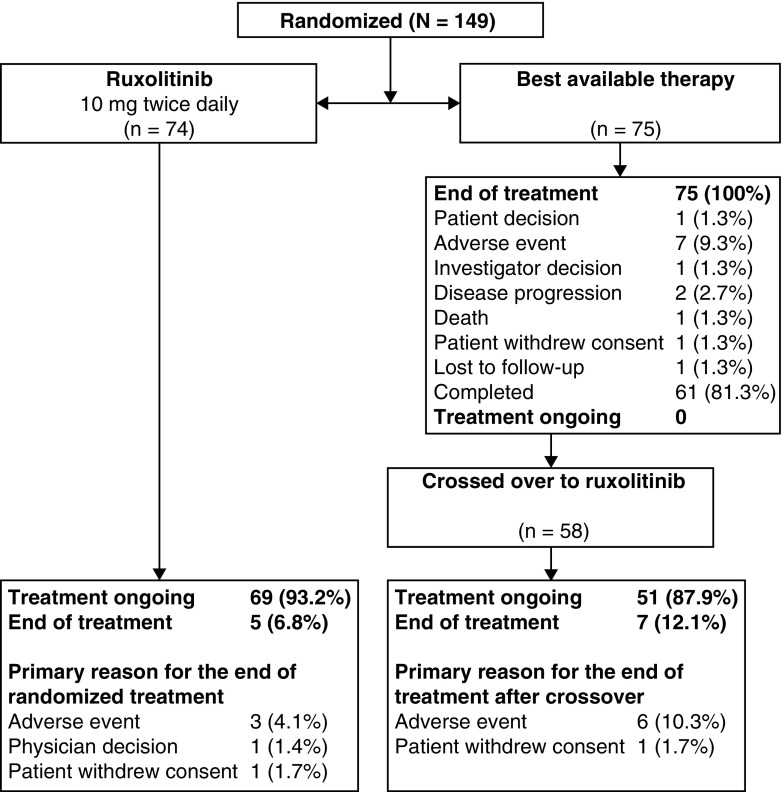
Patient disposition

At the time of data cutoff for the 80-week analysis, 69 patients (93.2%) randomized to the ruxolitinib arm were still receiving treatment (Fig. 1). The median duration of ruxolitinib exposure was 93.6 weeks (range 0.1–128.9 weeks). No patient was receiving BAT (median exposure, 28.4 weeks [range 6.7–83.0 weeks]) at the time of the week 80 analysis; 58 patients (77.3%) had crossed over to receive ruxolitinib therapy. Of those 58 patients, 51 (87.9%) continued to receive ruxolitinib at data cutoff (median exposure 60.1 weeks). Median dose intensity of ruxolitinib was 20.0 mg/day (interquartile range 19.5–27.2 mg/day). Among patients originally randomized to ruxolitinib, the most common reasons for discontinuation of the study drug were AEs (*n* = 3), including nonsmall cell lung cancer, metastases to lung, metastases to liver, and metastases to bone (*n* = 1); leukocytosis (*n* = 1); hypoesthesia and fatigue (*n* = 1); withdrawal of consent (*n* = 1); and physician decision (*n* = 1). Six of the seven patients who discontinued the treatment after the crossover were due to AEs (dyspepsia [*n* = 1]; peripheral vascular disorder [*n* = 1]; cholangiocarcinoma [*n* = 1]; thrombocytosis [*n* = 2]; or constipation [*n* = 1]).

### Efficacy

**Fig. 2:**
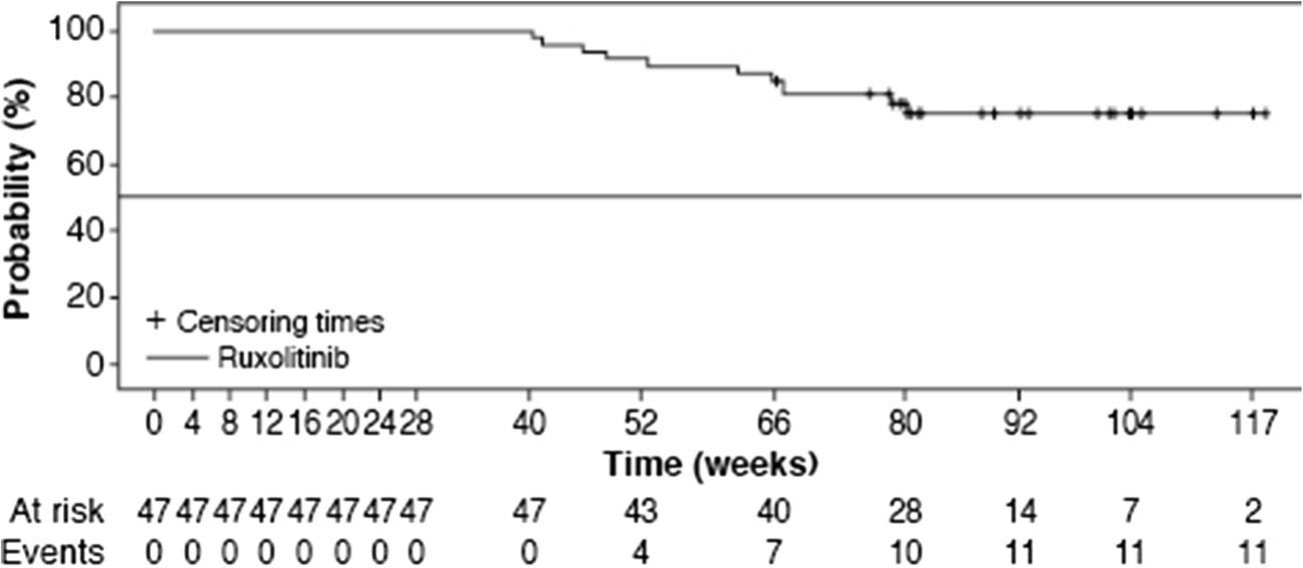
Duration of hematocrit control in patients who were originally randomized to ruxolitinib treatment

At the time of the primary analysis, HCT control was achieved in 62% (46/74) of ruxolitinib-treated patients compared to 19% (14/75) of patients treated with BAT therapy (odds ratio [OR] 7.28; 95% confidence interval [CI] 3.43–15.45; *P* < 0.0001) [17]. At week 80, one additional ruxolitinib-treated patient was found to be responding at week 28 (compared to the original analysis carried out with a data cutoff at week 28), thus increasing the total number of primary responders to 47 (64%). Among the patients who achieved an HCT response at week 28, the probability of maintaining the response up to week 80 was 78% in the ruxolitinib arm (Fig. 2). Overall, durable HCT control (defined as having HCT control at both week 28 and week 80) was achieved in 35/74 (47%) of ruxolitinib-treated patients (95% CI 35.6%, 59.3%) compared to 2/75 (3%) of patients treated with BAT (95% CI 0.3%, 9.3%) at week 80. The median duration of durable HCT control was not reached in either the ruxolitinib or BAT therapy arm. The estimated proportion of patients who were able to maintain the HCT control at 72 weeks was 81% in the ruxolitinib arm. Since the number of patients responding to BAT was very small, the proportion of patients who were able to maintain the response at 72 weeks in the BAT arm was not reported.

**Fig. 3:**
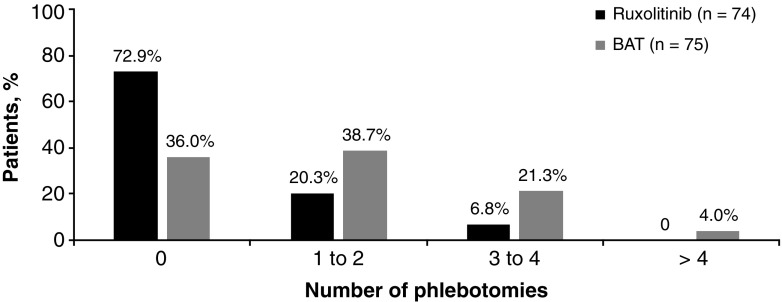
Number of phlebotomy procedures in ruxolitinib versus best available therapy arm (from baseline up to week 80). BAT, best available therapy

During the treatment period, there were fewer phlebotomies required to control HCT in patients who were randomized to ruxolitinib compared to patients randomized to BAT (36 versus 106 phlebotomies, respectively). In the ruxolitinib arm, 15 patients (20.3%) had 1–2 phlebotomies and 5 patients (6.8%) had 3–4 phlebotomies, with none having more than four phlebotomies. In the BAT arm, 29 patients (38.7%) had 1–2 phlebotomies, 16 patients (21.3%) had 3–4 phlebotomies, and 3 patients (4%) had more than 4 phlebotomies (Fig. 3). At week 80, the mean HCT decreased by 3.2% from baseline in ruxolitinib-treated patients (mean [standard deviation] HCT changed from 42.8% [1.46] at baseline to 39.6% [3.92] at week 80; *n* = 61), while it increased by 0.62% in the BAT arm (mean [standard deviation] changed from 42.4% [1.80] at baseline to 43.0% [3.02] at week 80; *n* = 6). Among the patients who crossed over from BAT to the ruxolitinib arm, the mean HCT values decreased as early as week 4 after crossover. At 52 weeks after the crossover, it decreased by 5.37% (mean [standard deviation] changed from 45.9% [3.64] at baseline to 40.5% [4.28] at week 80).

**Fig. 4:**
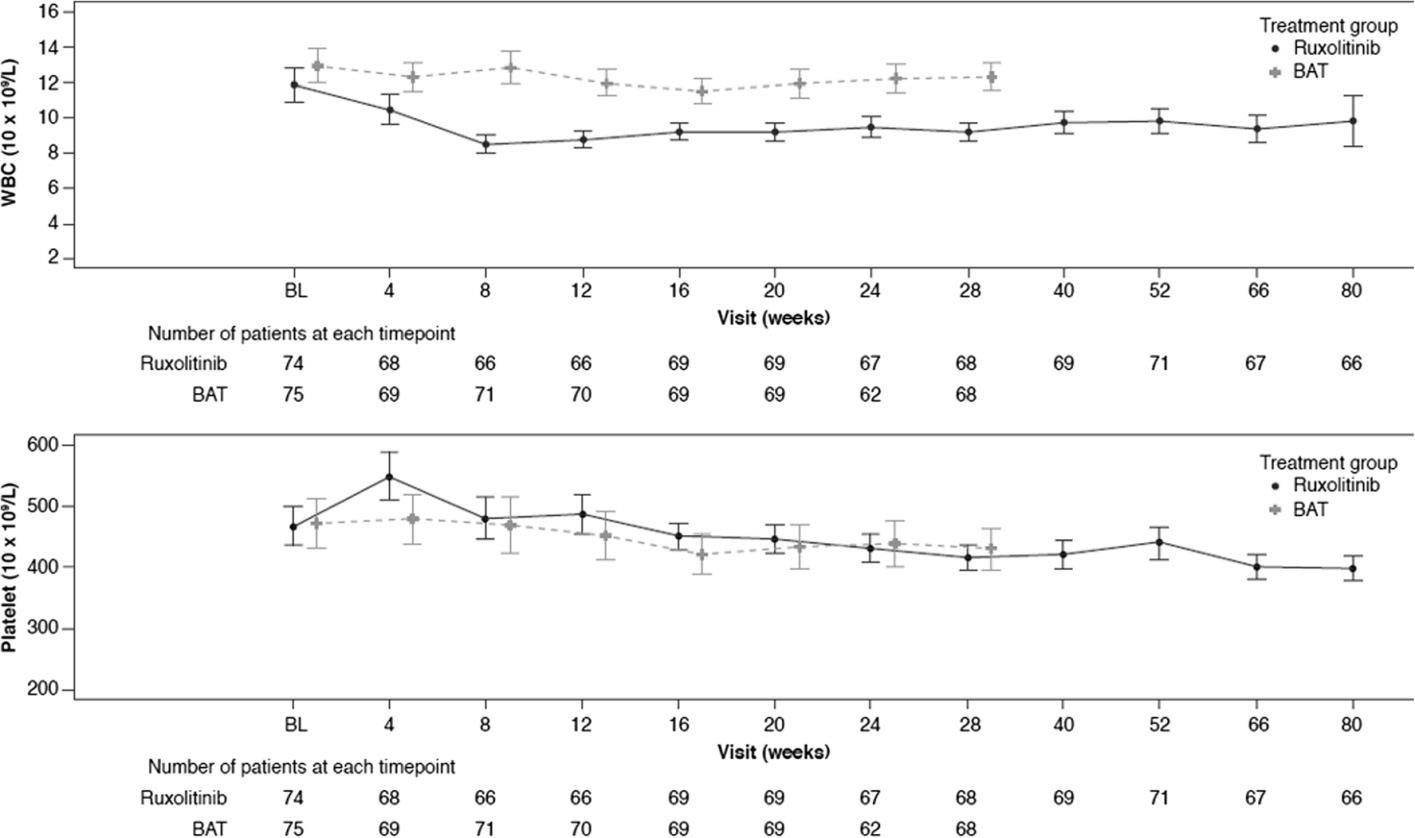
Mean white blood cell and platelet counts over time. BAT, best available therapy; WBC, white blood cells

A significantly higher proportion of patients originally randomized to ruxolitinib (18/74; 24.3%)—which included the one patient who was found to be a responder in the ruxolitinib arm after the primary analysis—achieved CHR at week 28 and continued through to week 80, compared to only 2/75 (2.7%) of BAT patients, resulting in an OR of 12.60 (95% CI 2.72, 58.44) in favor of ruxolitinib. At the time of the week 80 data cutoff, four patients (5.4%) in the ruxolitinib arm achieved partial remission based on the ELN and International Working Group-Myeloproliferative Neoplasms Research and Treatment (IWG-MRT) criteria, while none achieved partial remission in the BAT arm. Patients who were originally randomized to ruxolitinib had lower mean WBC counts compared to patients treated with BAT. At the time of this analysis, 69% of patients in the ruxolitinib arm had WBC counts < 10 × 10^9^/L and 51% had platelets counts ≤ 400 × 10^9^/L (Fig. 4). The median duration of CHR was 34 weeks in the ruxolitinib arm.

In general, patients treated with ruxolitinib reported greater improvement in the disease burden and overall quality of life as measured by MPN-SAF TSS, PSIS, EQ-5D-5L, WPAI, and PGIC scores, when compared to BAT. Consistent with the results from the week 28 cutoff, 45% of patients randomized to ruxolitinib demonstrated a ≥ 50% reduction in the MPN-SAF TSS at week 80 (Supplementary Fig. 1). This is also reflected by a sustained improvement (indicated by a negative mean change) in the total symptom scores observed in the ruxolitinib arm, with mean (standard deviation) change of − 9.0 (13.52) at week 80, consistent with what was observed at week 28 (− 10.46 [14.25]). At week 80, patients who were originally randomized to ruxolitinib showed an improvement in the majority of individual symptom scores, except abdominal discomfort and fever (Supplementary Fig. 2). As assessed by PSIS, 71% of patients treated with ruxolitinib showed improvement (“very much improved” and “much improved” responses) of pruritus through week 80 (Supplementary Fig. 3). Improvements in quality of life (QOL) measures, as evaluated by PGIC and EQ-5D-5L scores, were maintained with a longer term therapy of ruxolitinib (Supplementary Figs. 4 and 5). At week 80, 21/74 (28.4%) patients had at least one visit to the hospital in the ruxolitinib arm compared to 6/17 (35.3%) patients in the BAT arm who did not cross over to ruxolitinib. The mean *JAK2*V617F allele burden decreased consistently from baseline through week 80 for patients in the ruxolitinib arm (Supplementary Fig. 6); the mean (standard deviation) change from baseline in *JAK2*V617F allele burden (negative value indicates improvement) was − 4.7 (9.82) at week 28 and − 9.7 (16.10) at week 80. However, in the BAT arm, the data should be interpreted with caution since a significant number of patients crossed over to the ruxolitinib arm, reducing the number of evaluable patients to only 3 at week 80. The mean (standard deviation) change from baseline in the *JAK2*V617F allele burden, with only three evaluable patients, was − 2.0 (7.53) at week 28 and + 0.3 (5.04) at week 80. In the crossover patients, the mean (standard deviation) change from baseline in *JAK2*V617F allele burden after 52 weeks of crossover was − 9.0 (16.27).

### Safety

Long-term treatment with ruxolitinib was generally well tolerated, with 55% of patients in the ruxolitinib arm and 60% of patients who crossed over to ruxolitinib treatment not requiring dose reduction/interruption through week 80. The most common nonhematologic AEs (all grades) in the ruxolitinib arm were weight increase, arthralgia, and pruritus (Table 1). In the BAT arm, pruritus, headache, diarrhea, and upper respiratory tract infection were the most commonly reported all grade AEs. Among patients who crossed over to ruxolitinib from BAT, the AE rates and types were similar to those reported in the ruxolitinib group. In the crossover patient population, the most frequently reported nonhematological AEs were nasopharyngitis, headache, and hypertension. The overall rates of grade 3 or 4 AEs (21.1 versus 43.1 per 100 patient-years) and serious AEs (9.1 versus 16.9 per 100 patient-years) were lower in the ruxolitinib arm compared to the BAT arm. Hypertension (6.9%) was the most frequent grade 3 or 4 AE in patients who crossed over to the ruxolitinib arm. Among the hematologic AEs, the exposure-adjusted rates for AEs were lower in the ruxolitinib arm when compared to the BAT arm (thrombocytopenia [1.5 vs 15.0, respectively] and leukopenia [1.5 vs 1.9, respectively]) with the exception of anemia (14.3 per 100 patient-years in the ruxolitinib arm versus 3.7 per 100 patient-years in the BAT arm) (Table 2). Although the exposure-adjusted rates for anemia were higher with ruxolitinib, most events were mild to moderate in severity and did not lead to discontinuation of treatment.

**Table 1:** Nonhematologic adverse events in the 80-week analyses adjusted for exposure

Rate per 100 patient-years of exposure	Ruxolitinib (*n* = 74) exposure, patient-years^a^ = 132.59		Ruxolitinib crossover (*n* = 58) exposure, patient-years^a^ = 66.97		BAT (*n* = 75) exposure, patient-years^a^ = 53.36	
	All grades	Grade 3 or 4	All grades	Grade 3 or 4	All grades	Grade 3 or 4
Weight increased	10.6	0.8	6.0	0	1.9	0
Arthralgia	9.1	0	1.5	0	5.6	1.9
Pruritus	9.1	0	4.5	0	37.5	3.7
Constipation	8.3	0	7.5	1.5	7.5	0
Diarrhea	3.8	0	6.0	0	13.1	0
Nausea	3.0	0	4.5	0	9.4	0
Headache	8.3	0	9.0	0	16.9	0
Hypertension	8.3	6.8	9.0	6.0	5.6	5.6
Upper respiratory tract infection	2.3	0	3.0	0	13.1	0
Nasopharyngitis	3.8	0	9.0	0	3.7	0
Influenza	4.5	0.8	1.5	0	9.4	1.9
Asthenia	6.0	0.8	7.5	0	11.2	1.9
Fatigue	6.0	0.8	4.5	1.5	11.2	0
Dizziness	5.3	0	6.0	0	9.4	0
Night sweats	3.0	0	1.5	0	9.4	0
Decreased appetite	2.3	0	1.5	0	7.5	0

^a^Patient-year exposure is the sum of each patient’s exposure in days divided by 365.25. Adjusted rate for a given adverse event is calculated as number of events per 100 patient-years of exposure

Rate = n*100/(patient-year exposure)

**Table 2:** Hematologic adverse events in the 80-week analyses adjusted for exposure

Rate per 100 patient-years of exposure	Ruxolitinib (*n* = 74) exposure, patient-years^a^ = 132.59		Ruxolitinib crossover (*n* = 58) exposure, patient-years^a^ = 66.97		BAT (*n* = 75) exposure, patient-years^a^ = 53.36	
	All grades	Grade 3 or 4	All grades	Grade 3 or 4	All grades	Grade 3 or 4
Anemia	14.3	0	17.9	0	3.7	1.9
Hematoma	5.3	0	4.5	0	1.9	0
Thrombocytopenia	1.5	0	4.5	0	15.0	5.6
Hematocrit increased	0	0	0	0	9.4	1.9
Leukocytosis	3.8	0.8	3.0	1.5	7.5	1.9
Thrombocytosis	3.8	0	7.5	3.0	5.6	5.6

^a^Patient-year exposure is the sum of each patient’s exposure in days divided by 365.25. Adjusted rate for a given adverse event is calculated as number of events per 100 patient-years of exposure

Rate = n*100/(patient-year exposure)

*BAT*, best available therapy

Since the week 28 analysis, 2 new patients in the ruxolitinib group had thromboembolic events. Of these 2 patients, one had retinal vascular thrombosis and the other had thrombophlebitis. Both the events were found to be of grade 2 severity (Table 3). At week 80, the rates of all grade and grade 3 or 4 hemorrhage events per 100 patient-years of exposure were 9.8 and 1.5, respectively, among patients originally randomized to ruxolitinib, versus 15.0 and 1.9 in those receiving BAT. The rates of all grade and grade 3 or 4 infections per 100 patient-years of exposure were 24.9 and 2.3, respectively, in the ruxolitinib arm, versus 33.7 and 3.7 in those receiving BAT. Rates of herpes zoster infection were higher in patients receiving ruxolitinib; all grade exposure-adjusted rates of herpes zoster infection (per 100 patient-years of exposure) were 3.8 in patients originally randomized to ruxolitinib, 7.5 in patients receiving ruxolitinib after crossover, and none in the BAT arm (Table 4). At the time of the week 28 analysis, there was no patient in the ruxolitinib arm that had any drug-related serious AE. However, after the week 28 cutoff, squamous cell carcinoma of the skin was reported in one patient treated with ruxolitinib. On performing the histology of the other body regions for this patient, the data showed presence of intra-epidermal carcinoma, basal cell carcinoma, and solar keratosis. In patients originally randomized to ruxolitinib and patients who crossed over to ruxolitinib, most of the hematology and biochemistry abnormalities were of grade 1 or 2 in severity.

**Table 3:** Thromboembolic events in the 80-week analysis adjusted for exposure

Rate per 100 patient-years of exposure	Ruxolitinib (*n* = 74) exposure, patient-years^b^ = 132.59		Ruxolitinib crossover (*n* = 58) exposure, patient-years^b^ = 66.97		BAT (*n* = 75) exposure, patient-years^b^ = 53.36	
	All grades	Grade 3 or 4	All grades	Grade 3 or 4	All grades	Grade 3 or 4
Total events^a^	1.5 (2)	0 (0)	0 (0)	0 (0)	1.9 (1)	0 (0)
Retinal vascular thrombosis	0.8 (1)	0 (0)	0 (0)	0 (0)	0 (0)	0 (0)
Thrombophlebitis^c^	0.8 (1)	0 (0)	0 (0)	0 (0)	1.9 (1)	0 (0)

^a^Embolic and thrombotic events (SMQ) regardless of study-drug relationship by preferred term, maximum grade, and treatment group

^b^Patient-year exposure is the sum of each patient’s exposure in days divided by 365.25. Adjusted rate for a given adverse event is calculated as number of events per 100 patient-years of exposure. Rate = n*100/(patient-year exposure)

^c^Thrombophlebitis (one event) was reported in the ruxolitinib arm and superficial thrombophlebitis (one event) was reported in the BAT arm

All patients were on low-dose aspirin (75–150 mg/day), unless medically contraindicated

*BAT*, best available therapy; *MedDRA*, Medical Dictionary for Regulatory Activities; *SMQ*, standardized MedDRA query

**Table 4:** Adverse events of interest in the 80-week analysis adjusted for exposure

Rate per 100 patient-years of exposure	Ruxolitinib (*n* = 74) exposure, patient-years^a^ = 132.6		Ruxolitinib crossover (*n* = 58) exposure, patient-years^a^ = 66.97		BAT (*n* = 75) exposure, patient-years^a^ = 53.36	
	All grades	Grade 3 or 4	All grades	Grade 3 or 4	All grades	Grade 3 or 4
Infections and infestations	24.9	2.3	29.9	1.5	33.7	3.7
Herpes zoster infection	3.8	0	7.5	0	0	0
Urinary tract infection	1.5	0	1.5	0	0	0
Pneumonia	0.8	0	0	0	1.9	1.9
Urosepsis	0.8	0	0	0	0	0
Septic shock	0	0	0	0	1.9	1.9
Neoplasms: benign, malignant and unspecified	6.8	3.0	4.5	1.5	13.1	9.4
Malignant melanoma^b^	0.8	0.8	0	0	0	0
Squamous cell carcinoma of skin^b^	0.8	0.8	0	0	0	0
Squamous cell carcinoma	0	0	3.0	0	1.9	1.9
Myelofibrosis	0	0	0	0	1.9	1.9
Acute myeloid leukemia	0	0	0	0	1.9	1.9

^a^Patient-year exposure is the sum of each patient’s exposure in days divided by 365.25. Adjusted rate for a given adverse event is calculated as number of events per 100 patient-years of exposure. Rate = n*100/(patient-year exposure)

Rates of overall secondary malignancies per 100 patient-years of exposure were 6.8 in those originally randomized to ruxolitinib, 4.5 with ruxolitinib after crossover, and 13.1 with BAT (Table 4). The rates of nonmelanoma skin cancer (NMSC) were 0.8 in those originally randomized to ruxolitinib, 1.5 with ruxolitinib after crossover, and 1.9 with BAT. No patients in the ruxolitinib arm or those who crossed over to ruxolitinib had transformation to MF or AML. In the BAT arm, transformation to MF and AML occurred in 1 patient each (rate of transformation 1.9 per 100 patient-years of exposure). During the study, there were no deaths in the ruxolitinib arm or crossover patient population. In the BAT arm, 1 additional death was reported since the week 28 data cutoff. The patient died after the treatment had ended due to the progression of PV to AML. The death was considered unrelated to treatment.

## Discussion

The results from the long-term follow-up analysis at week 80 of the RESPONSE-2 study further add to the evidence for the efficacy and safety profile of ruxolitinib in providing durable control of HCT and durable peripheral blood count remission in HU-resistant/intolerant PV patients with a nonpalpable spleen. At the 80-week analysis, a substantial proportion of patients originally randomized to ruxolitinib had maintained HCT control. The majority of patients (78%) in the ruxolitinib arm who achieved the HCT response at week 28 were predicted to maintain the response at week 80. This is consistent with the RESPONSE study, where the probability of maintaining HCT control in the ruxolitinib arm for at least 80 weeks from time of response was 89% [14]. Not surprisingly, patients who crossed over to receive ruxolitinib treatment after week 28 demonstrated a similar improvement in mean HCT levels at week 80 as did patients who were originally randomized to ruxolitinib. Of note is that approximately 73% of patients in the ruxolitinib arm did not require phlebotomy over the course of treatment compared to 36% in the BAT arm. The total number of phlebotomies was 3 times higher in the BAT arm compared to that in the ruxolitinib arm, leading to an increase in healthcare utilization, and potential adverse consequences of iatrogenic iron deficiency. Similarly, CHR was durable in a higher proportion of patients in the ruxolitinib arm compared to the BAT arm.

Importantly, improvements were consistently seen in the other efficacy end points in patients treated with ruxolitinib. In this study, a substantially higher proportion of patients in the ruxolitinib arm continued to show improvement in symptom burden and QoL on a longer follow-up. In contrast, the majority of cytoreductive therapies, including the first-line HU, are associated with severe toxicity profiles that are known to compromise patients’ QoL [3]. Similarly, the treatment of PV with phlebotomy might also need a reconsideration when the symptoms due to iron deficiency start interfering in patient’s QoL [19]. Although the importance of HCT control in the prevention of thrombotic events has been established [8], the effect of HCT control on symptom improvement and QoL is not well characterized and needs further exploration [9]. The results from both RESPONSE and RESPONSE-2 studies demonstrate that ruxolitinib provided HCT control and improved symptom burden and QoL in patients with PV.

A sustained reduction in the *JAK2*V617F allele burden through week 80 was observed in patients who were randomized to ruxolitinib and those who crossed over to ruxolitinib after week 28, but not in patients who received BAT. These allele burden data also suggest that patients who might not have achieved a molecular response with BAT were able to obtain modest reductions in allele burden after crossing over to ruxolitinib treatment. However, the implication of allele burden reduction on clinical benefits is not well understood and warrants further exploration. In an exploratory analysis from the RESPONSE study, the treatment of patients with ruxolitinib for up to 4 years provided progressive reductions in the *JAK2*V617F allele burden [20]. Greater spleen volume reduction was observed in patients with pronounced reduction in the allele burden. Other than spleen volume, no other hematological parameter (HCT, WBC, or platelet count) showed an association with the *JAK2*V617F allele burden reduction in this analysis [20].

The safety data from this long-term week 80 analysis are consistent with the previous studies [13, 14, 17]. Given its mechanism of action as a JAK1 and JAK2 inhibitor, the most common AEs associated with ruxolitinib were anemia and thrombocytopenia. However, most events recorded at week 80 in the RESPONSE-2 study were of grade 1 or 2 severity and rarely led to treatment discontinuation. The rates of overall and grade 3 or 4 infections were generally lower in patients treated with ruxolitinib compared to BAT, except an increased frequency of herpes zoster infections in the ruxolitinib arm. As reported in a recent publication by Polverelli et al. (2017), these infections could be better controlled in real-world patients by employing active screening techniques, comprehensive patient education, and prophylactic or rapid access to treatment at symptom onset [21]. The incidence of NMSC was generally similar between ruxolitinib- and BAT-treated patients. One patient in the ruxolitinib arm developed squamous cell carcinoma of the skin. In addition, 1 patient in the crossover group, who had a history of pre-basal cell carcinoma lesion on the nose at baseline, developed basal cell carcinoma. Since HU treatment is reported to be associated with the development of precancerous skin lesions and NMSC [22], the previous therapy with HU in these patients could be a contributor of the NMSC. Of note, no patient in the ruxolitinib arm had disease progression, compared with 2 patients in the BAT arm. The long-term data from 260 weeks of follow-up is expected to be more conclusive in this regard. Although treatment with ruxolitinib was associated with durable HCT control and reduction in leukocytes (markers for reductions in thrombotic events and improvements in survival), the study was not designed to evaluate reduction in thrombotic risk. Overall, no new safety signals have been observed with this long-term analysis compared with the week 28 analysis.

In conclusion, ruxolitinib provided durable HCT control, durable CHR, reduction in phlebotomy requirement, improved symptom burden, and continuous reduction in JAK2 allele burden, and was well tolerated with more than 90% of patients receiving treatment at week 80 of the RESPONSE-2 study. The long-term data from the RESPONSE-2 study are consistent with the findings from the RESPONSE study, and hence add further to the evidence that ruxolitinib should also be considered as a standard of care in patients who are refractory or resistant to hydroxyurea.

## Electronic supplementary material

ESM 1 (DOCX 357 kb)
