# Supplementary material for: Ruxolitinib for the treatment of inadequately controlled polycythemia vera without splenomegaly: 80-week follow-up from the RESPONSE-2 trial
Source: Ann Hematol. 2018 May 27;97(9):1591–600. doi: 10.1007/s00277-018-3365-y (PMC6097748; doi:10.1007/s00277-018-3365-y)
Supplement: Supplementary file 1 — (DOCX 357 kb) [file 277_2018_3365_MOESM1_ESM.docx]

**Supplementary appendix**

**Ruxolitinib for the treatment of inadequately controlled polycythemia vera without splenomegaly: 80-week follow-up from the RESPONSE-2 trial**

**Martin Griesshammer,^1^ Guray Saydam,^2^ Francesca Palandri,^3^ Giulia Benevolo,^4^ Miklos Egyed,^5^ Jeannie Callum,^6^ Timothy Devos,^7^ Serdar Sivgin,^8^ Paola Guglielmelli,^9^ Caroline Bensasson,^10^ Mahmudul Khan,^11^ Julian Perez Ronco,^12^ Francesco Passamonti^13^**

^1^Department of Hematology, Oncology, Hemostaseology and Palliative Care, Johannes Wesling Clinic, Minden, Germany; ^2^Department of Hematology, Ege University Medical Faculty, Izmir, Turkey; ^3^Department of Hematology/Oncology, Seràgnoli Institute of Hematology, Bologna University School of Medicine, Bologna, Italy; ^4^Department of Hematology, Città della Salute e della Scienza di Torino, Turin, Italy; ^5^Hematology Department of Somogy County, Kaposi Mor Teaching Hospital, Kaposvar, Hungary; ^6^Department of Transfusion Medicine and Tissue Banks, Sunnybrook Health Sciences Centre, Toronto, Ontario, Canada; ^7^Department of Hematology, University Hospitals Leuven and Laboratory of Experimental Transplantation, Department of Microbiology and Immunology, KU Leuven, Leuven, Belgium; ^8^Department of Hematology, Dedeman Stem Cell Transplantation Hospital, Erciyes University, Kayseri, Turkey; ^9^CRIMM, Center for Research and Innovation of Myeloproliferative Neoplasms, AOU Careggi, Department of Experimental and Clinical Medicine, University of Florence, Florence, Italy; ^10^Novartis Pharma S.A.S., Rueil-Malmaison, France; ^11^Novartis Pharmaceuticals Corporation, East Hanover, New Jersey, USA; ^12^Novartis AG, Basel, Switzerland; ^13^Department of Hematology, University of Insubria, Varese, Italy

**Correspondence:**

Professor Dr. Martin Griesshammer

Director,

Department of Hematology, Oncology, Hemostaseology and Palliative Care,

Johannes Wesling Clinic,

Minden, Germany

Telephone: 0571 790-4201

Fax: 0571 790-294200

Email: [martin.griesshammer@muehlenkreiskliniken.de](mailto:martin.griesshammer@muehlenkreiskliniken.de)

**Statistical Analysis**

A total of 116 patients were needed to detect a significant difference between treatment groups with a two-sided t-test at the significance level of 0.05 and 90% power. The efficacy analysis for the primary and key secondary endpoints was carried out according to an intention-to-treat principle, including data from all patients randomly assigned to treatment. Patients with missing assessments that prevented investigation of the primary and secondary endpoints were regarded as non-responders. Other secondary efficacy endpoints were not alpha controlled, and statistical tests were done for descriptive purposes only and were not adjusted for multiple comparisons. Symptom assessments included all patients with available baseline symptom measures.


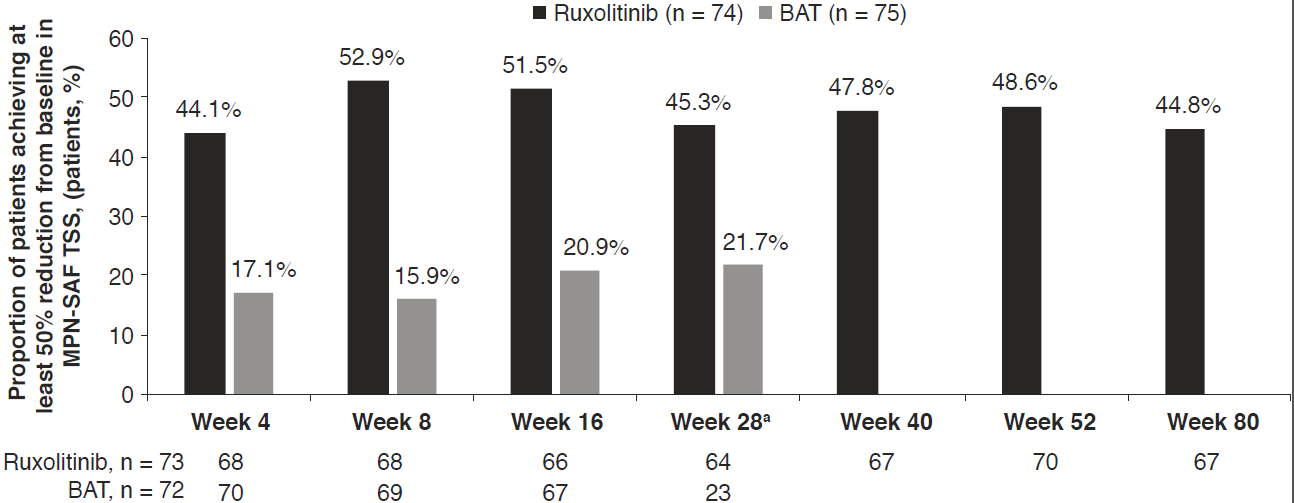


^a^Patients randomly assigned to BAT crossed over to ruxolitinib from Week 28 if they did not meet the primary endpoint or later if treatment was shown to be ineffective (i.e., HCT level > 45%, or if they received phlebotomy) or for safety-related reasons.

BAT, best available therapy; HCT, hematocrit; MPN-SAF TSS, Myeloproliferative Neoplasm Symptom Assessment Form Total Symptom Score.

**Supplementary Figure 1. Proportion of patients with at least a 50% reduction in the total symptom score over time**


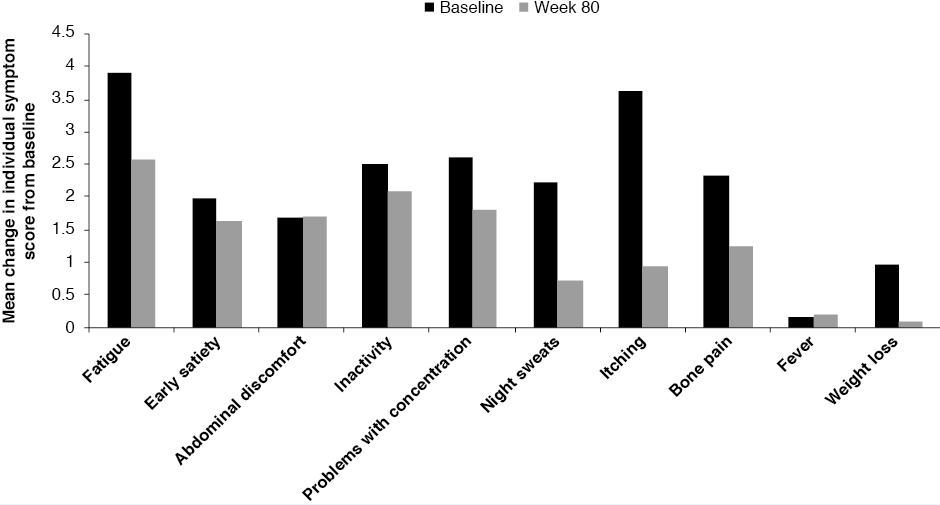


**Supplementary Figure 2. The change in the individual symptom scores from baseline at week 80 in patients who were originally randomized to ruxolitinib**


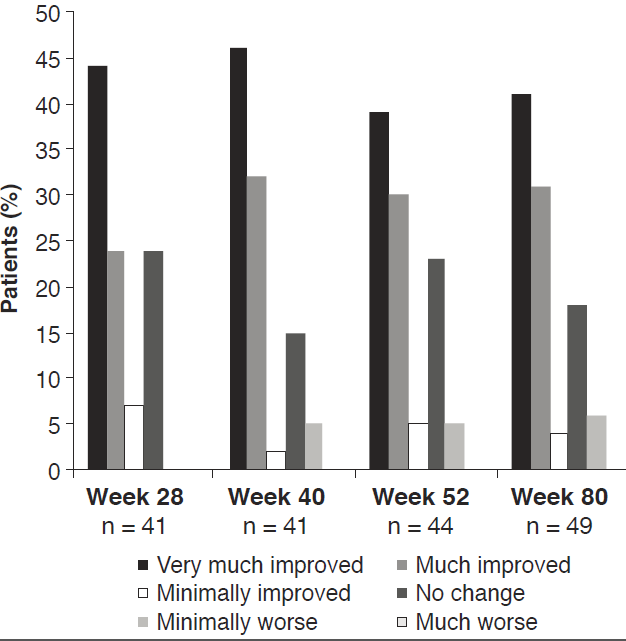


**Supplementary Figure 3. Pruritus Symptom Impact Scale over time in patients originally randomized to ruxolitinib**


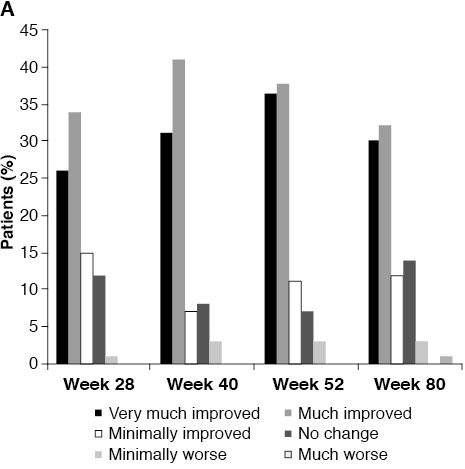

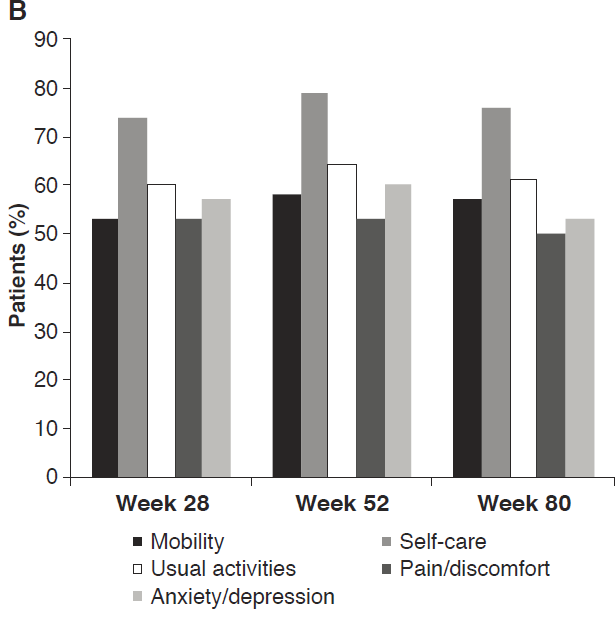


**Supplementary Figure 4. Patient Global Impression of Change over time** **in patients originally randomized to ruxolitinib (A), and the proportion of patients reporting no problems in the individual domains of the EuroQol-5D-5L over time in patients originally randomized to ruxolitinib (B).**


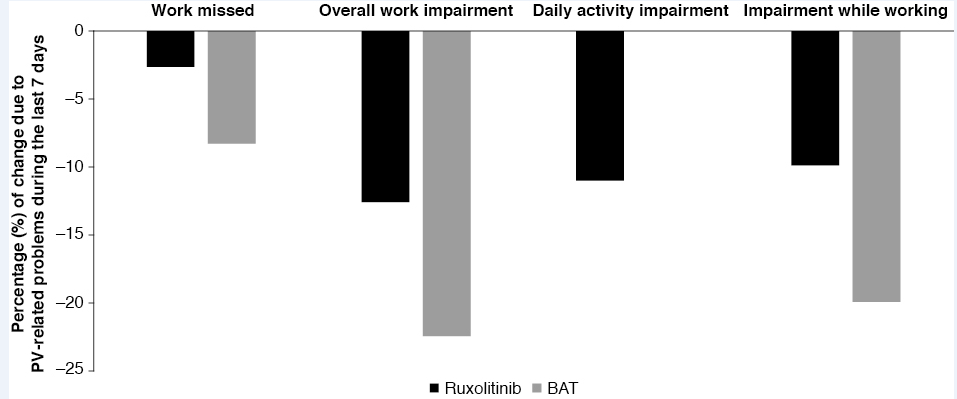


BAT, best available therapy; PV, polycythemia vera

**Supplementary Figure 5. Mean change from baseline in the Work Productivity and Activity Impairment questionnaire at week 52**


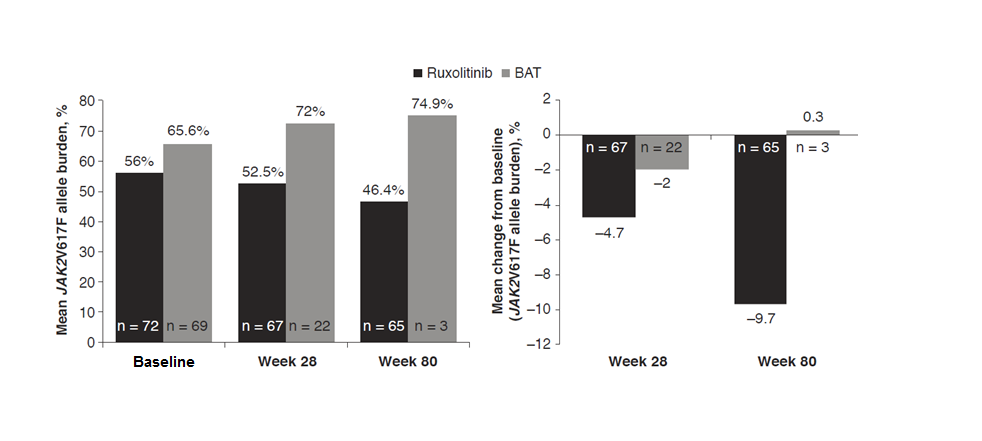


*Crossover data is not presented in the figure.

BAT, best available therapy; JAK, Janus kinase

**Supplementary Figure 6. Mean change from baseline in *JAK2*V617F allele burden**
